# The UCI Phonotactic Calculator: An online tool for computing phonotactic metrics

**DOI:** 10.3758/s13428-025-02725-z

**Published:** 2025-07-03

**Authors:** Connor Mayer, Arya Kondur, Megha Sundara

**Affiliations:** 1 University of California Irvine, Irvine, CA USA; 2 University of California Los Angeles, Los Angeles, CA USA

**Keywords:** Phonology, Phonotactics, Acceptability judgments, Speech, Language

## Abstract

This paper presents the UCI Phonotactic Calculator (UCIPC), a new online tool for quantifying the occurrence of segments and segment sequences in a corpus. This tool has several advantages compared to existing tools: it allows users to supply their own training data, meaning it can be applied to any language for which a corpus is available; it computes a wider range of metrics than most existing tools; and it provides an accessible point-and-click interface that allows researchers with more modest technical backgrounds to take advantage of phonotactic models. After describing the metrics implemented by the calculator and how to use it, we present the results of a proof-of-concept study comparing how well different types of metrics implemented by the UCIPC predict human responses from eight published nonce word acceptability judgment studies across four different languages. These results suggest that metrics that take into account the relative position of sounds and include word boundaries are better at predicting human responses than those that are based on the absolute position of sounds and do not include word boundaries. We close by discussing the usefulness of tools like the UCIPC in experimental design and analysis and outline several areas of future research that this tool will help support.

**Supplementary Information:**

The online version contains supplementary material available at 10.3758/s13428-025-02725-z.

## Introduction

*Phonotactics* refers to restrictions on the sequencing of sounds into words. For example, although the word /skif/ “skeef” is not a real English word (at least in the authors’ dialects), it could in principle become an English word. It could not be a Spanish word, however, because there are no Spanish words that begin with /s/-initial complex onsets. The fact that different languages impose different restrictions on phonotactic patterns indicates that phonotactics must be learned from speech input (though some aspects like sonority sequencing preferences have been proposed to be innate, e.g., Berent et al., 2008; Prince & Smolensky, 1993; Selkirk, 1984). Speakers generally have strong intuitions about what possible words could sound like in their language. The process of developing these intuitions is typically taken to correspond to forming generalizations over sound patterns in the lexicon, with learners forming generalizations based on the type frequency (rather than token frequency) of particular phonotactic structures (e.g., Chomsky & Halle, 1965, 1968; Bybee, 1995, 2003; Pierrehumbert, 2001; Bailey & Hahn, 2001; Edwards et al., 2004, a.o.).

One common method for gaining insight into speakers’ phonotactic knowledge is to perform acceptability judgment tasks where speakers are asked to rate a nonce word based on its suitability as a possible word in their language. This might involve a forced-choice task, a numeric rating, or other responses like magnitude estimation. It has long been observed on the basis of such studies that phonotactic judgments are graded: speakers do not generally think of words as being “in” or “out” but can often arrange them on a cline of acceptability. The classic example from Chomsky and Halle (1968) is the three words “blick,” “bnick,” and “bnzk.” Although none of these is a real English word, speakers typically find “blick” to be acceptable, “bnzk” to be unacceptable, and “bnick” to be somewhere in between. Every experimental study that has tested for gradience has found it (e.g., Coleman & Pierrehumbert, 1997; Scholes, 1966; Bailey & Hahn, 2001; Hayes & Wilson, 2008; Daland et al., 2011, a.o.).

Further, graded knowledge of phonotactics is crucially important for speech perception (e.g., Norris & McQueen, 2008; Davidson & Shaw, 2012, Dupoux et al., 2011, Chodroff & Wilson, 2014; Steffman & Sundara, 2024) and speech production (e.g., Edwards et al., 2004). It is also important for word segmentation and word learning, even in infants and children (e.g., Mattys et al., 1999; McQueen, 1998; Mersad & Nazzi, 2011; Vitevitch & Luce, 1999; Storkel, 2001). Speech errors produced by native speakers also respect gradient phonotactic restrictions (e.g., Goldrick & Larson, 2008; Taylor & Houghton, 2005; Warker, 2013; Warker & Dell, 2006, 2015). As researchers, we are interested in understanding what knowledge of phonotactics speakers possess, how they acquire that knowledge, and how it is deployed in other areas of language. A common approach is to develop models that allow phonotactic metrics to be computed for words given some training data sample meant to approximate a speaker’s lexicon, over which phonotactic generalizations can be formed. These metrics are commonly used as predictors of experimental data such as reading time, categorization, accuracy or reaction time for lexical decisions, production, speech errors, or acceptability scores. Such models are useful because they allow us to stipulate precisely (a) what phonotactic configurations speakers are sensitive to, (b) how exposure to these configurations shapes phonotactic knowledge, and (c) how phonotactics influences speech more generally. A key criterion for these models is that they output graded rather than categorical acceptability scores.

The goal of this paper is to present a new online tool used for calculating phonotactic metrics: the University of California, Irvine (UCI) Phonotactic Calculator (https://phonotactics.socsci.uci.edu/). This tool has several advantages compared to existing tools for computing graded lexical statistics: it allows users to supply their own training data, meaning it can be run on any language where a corpus representing the lexicon is available; it computes a wider range of metrics than is typical; and it provides an accessible point-and-click interface that allows researchers with more modest technical backgrounds to take advantage of phonotactic models.

The paper is structured as follows: Sect. "Limitations of existing tools for computing phonotactic acceptability" describes several existing tools for computing phonotactic metrics and the limitations of these tools that motivated the development of the UCI Phonotactic Calculator. Sect. "The UCI Phonotactic Calculator" describes the UCI Phonotactic Calculator and the metrics it implements. Sect. "Applications of phonotactic metrics in experiments with adults" shows how the UCI Phonotactic Calculator can be used to compare a variety of proposed phonotactic models against data from eight phonotactic acceptability studies across four languages. The results demonstrate that, in every case, metrics that reference the relative position of segments in words outperform more commonly used metrics that reference the absolute position of segments. This raises several other questions that would benefit from future research. Sect. "Applications of phonotactic metrics in stimulus construction" offers a brief discussion and conclusion.

## Limitations of existing tools for computing phonotactic acceptability

In this section we will discuss existing tools that quantify the occurrence of a segment or segment sequences in a corpus embodying a lexicon. Overall, such tools are available for a small number of languages and cannot be customized because the training corpora they are based on are not accessible to the user. Both these limitations severely restrict the range of questions that can be empirically investigated.

### The Phonotactic Probability Calculator

Currently, the most well-known phonotactics calculator, with 675 citations in Google Scholar, is the Phonotactic Probability Calculator (PPC; Vitevitch & Luce, 2004). The PPC allows the phonotactic metrics described in Jusczyk et al. (1994) and Vitevitch and Luce (1999) to be computed for novel words in English, Spanish, and Modern Standard Arabic (Aljasser & Vitevitch, 2018). A related tool, the Neighborhood Density Calculator, allows neighborhood density to be calculated for words in the same languages (Vitevitch & Luce, 2016).

The English model is trained on a phonetic transcription of the 1964 Merriam-Webster Pocket Dictionary, which consists of about 20,000 words. Information about word frequency comes from Kučera and Francis (1967). Stress is not encoded. The website does not state what training data were used for the Spanish model, though it may be the data from the Beginning Spanish Lexicon (Vitevitch et al., 2012), which consists of 3,854 words from the glossary of a first-year Spanish textbook, transcribed in Castilian Spanish. The Modern Standard Arabic model is trained on a list of the 100,000 most frequent Modern Standard Arabic lemmas purchased from https://www.sketchengine.eu/.

The metrics calculated by the PPC are *absolute positional*, *frequency-weighted* *unigram* and *bigram scores.* The mathematical implementation of this is described in detail in Sect. "Absolute positional bigram metrics", but we provide some intuitive definitions of the properties of these metrics here:

- *Unigram*/*bigram:* Unigram metrics consider the frequency of occurrence of individual sounds. Bigram metrics consider the frequency of occurrence of adjacent pairs. Together these measures can capture how likely listeners are to hear individual sounds, as well as particular sequences of sounds.
- *Frequency-weighted:* Words that have a higher token frequency contribute more than do less frequent words. In a model that is not frequency-weighted, all word types in the training set contribute equally, regardless of their token frequency.
- *Absolute positional:* The absolute position of the unigrams/bigrams in a word is considered when calculating metrics*.* This differs from standard unigram/bigram models, where absolute position is not considered (e.g., Bahl et al., 1983; Chen & Goodman, 1999; Jurafsky & Martin, 2025; Markov, 1913; Shannon, 1948). For example*,* in an *absolute positional* unigram model, the influence of a /t/ on the computed metric for a word can differ depending on whether it occurs in the first position, second position, and so on. In a *relative positional* unigram model, to be described below, the influence of /t/ on the metric will be the same regardless of where in the word it occurs. Analogously, in an absolute positional bigram model, the influence of a sequence /st/ on the computed metric can differ depending on whether this sequence occurs as the first and second sounds, the second and third, etc. In a relative positional bigram model, the influence of this sequence on the metric will be the same regardless of the positions it occurs in. The term “relative positional” is meant to indicate that the model can make reference only to the position of a sound in a word relative to other sounds (e.g., does /t/ occur following an /s/), while in an absolute positional model, the model can additionally make reference to the specific position in a word in which a sound or sequence occurs. In more rigorous mathematical terms, absolute positional models are *nonstationary,* while the relative positional models are *stationary* or *translation-invariant*.

The absolute positional metrics implemented in Vitevitch and Luce (2004) have two other important differences from standard implementations of relative positional metrics. First, the absolute positional metrics do not explicitly reference word boundaries. This is not an issue for word-initial segments, since these are always in the first position, but it means these metrics cannot differentiate between segments that occur at the end of words and segments that do not. In relative positional models, word boundary symbols are typically inserted at the edges of words and thus referenced when computing the metrics (so, for example, a bigram sequence like /t#/, where # is a word boundary, refers to a /t/ in word-final position). Second, the absolute positional bigram metrics are implemented as joint probabilities ($P\left(A\;and\;B\right)$) while the relative positional metrics are implemented as conditional probabilities ($P\left(B|A\right)$). These issues will be discussed more in Sect. "The UCI Phonotactic Calculator".

Although this model has been extremely influential, it has a number of limitations, both mathematical and practical. We will discuss the mathematical issues in Sect. "Currently supported phonotactic metrics". In terms of practical issues, because training data are hardcoded into the PPC, it is available only for English, Spanish, and Modern Standard Arabic, and the properties of the training data cannot be customized. Finally, the use of absolute positional unigram and bigram metrics may lead to data sparsity issues for longer words. For example, the mean number of phonemes in an English word is about 5.77 (*SD* = 1.93; Marian et al., 2012). Estimates for a unigram score corresponding to /t/ in the third position will be based on a large number of data points (since most English words have something in the third position), while estimates for a score corresponding to /t/ in the 10th position will be less reliable, as fewer words have 10 or more segments. This is not an issue for relative positional models, because unigram and bigram values are calculated without taking absolute position into account.

### Irvine Phonotactic Online Dictionary

A tool called the Irvine Phonotactic Online Dictionary (IPhOD; Vaden et al., 2010; http://www.iphod.com/) provides similar functionality to the PPC. IPhOD can compute a large range of phonotactic metrics, including both relative/absolute positional and frequency-weighted/non-frequency-weighted metrics, as well as neighborhood densities for English words provided by the user. In addition, it allows users to search for words that meet certain criteria with respect to these metrics (e.g., “find English words with fewer than three neighbors”).

There are two main limitations of the IPhOD calculator. The first is that the training dataset is limited to English, and more specifically to the approximately 54,000 words in the Carnegie Mellon University (CMU) English Pronouncing Dictionary (Weide, 1994). While this database is quite extensive, words that do not exist in it cannot be used as part of the calculation process. This also limits users’ ability to provide their own training dataset, which may be more practical for certain research purposes. Second, the overall usage of the IPhOD calculator is limited, as it only supports a few phonotactic metrics, and users must enter their testing dataset manually rather than through a file upload. These are minor issues that we aim to address with the UCI Phonotactic Calculator.

### CLEARPOND

CLEARPOND is a tool maintained by Northwestern University that computes orthographic and phonological neighborhood density and other metrics like word length and frequency (Marian et al., 2012; https://clearpond.northwestern.edu/). CLEARPOND supports five languages—English, Dutch, French, German, and Spanish—and also allows cross-language neighborhood densities to be computed. CLEARPOND also supports the calculation of absolute positional, frequency-weighted biphone and bigram scores using the same method as described in Vitevitch and Luce (2004). Unlike the other programs described here, CLEARPOND has experimental functionality that computes neighborhood density and neighbors for a list of training words given a custom training dataset. This functionality is limited in that it only computes a subset of the neighborhood density metrics and no phonotactic metrics. Thus, CLEARPOND is similar to the PPC but with support for a wider range of languages.

### The UCLA Phonotactic Learner

The University of California, Los Angeles (UCLA) Phonotactic Learner (Hayes & Wilson, 2008; https://linguistics.ucla.edu/people/hayes/Phonotactics/) is a program for calculating phonotactic probabilities. It is a maximum entropy model (Goldwater & Johnson, 2003) that penalizes words that violate certain *featural n-gram constraints.* Features refer to properties of sounds like voicing, sonority, and manner of articulation. Examples of such constraints might be “don’t have a voiced sound following a voiceless sound.” The UCLA Phonotactic Learner induces from a training set both what constraints are necessary and how strongly they should be weighted.

An advantage of referring to features rather than segments is that it can capture variability in the acceptability of unattested sequences. For example, even though both “bnick” and “bnzck” are poorly formed with respect to English phonotactics, speakers often intuitively perceive that the first is not as bad as the second (Chomsky & Halle, 1968). Models that refer to segments alone, like all the models discussed above, cannot capture this distinction, since both /bn/ and /bz/ are unattested. Featural models, however, can capture the idea that because there are more onsets like /bn/ (e.g., onsets with a /b/ followed by non-nasal coronal sonorant, like /bl/, or an obstruent followed by a coronal nasal, like /sn/) than there are like /bz/, speakers should find the former more acceptable.

The UCLA Phonotactic Learner has been an enormously influential model of phonotactic learning, with over 1,000 citations on Google Scholar at the time of this writing, and its performance compares favorably to other models (e.g., Daland et al., 2011). Some limitations are that it is a standalone executable and cannot integrate directly into programming workflows, and that it does not output probabilities directly but rather numeric weights that correlate with them. The model has the additional task relative to the other models of discovering the constraints: there are several hyperparameters that govern how this process takes place that the model is sensitive to.

### Other tools

There are also several databases that contain phonotactic or neighborhood density metrics for individual languages. EsPal allows neighborhood densities and other metrics to be calculated for both Latin American and Castilian Spanish based on both written and spoken corpora, and supports relative/absolute positional and frequency-weighted/non-frequency-weighted metrics, as well as neighborhood densities (Duchon et al., 2013). It also allows Spanish words to be selected based on these properties. Diphones-fr is a simple database that contains diphone frequency information from over 50 million French words (New & Spinelli, 2013).

The remaining existing tools are mainly used for word selection or generation under restrictions on phonotactic probability or neighborhood density. In this sense, they do not provide the same functionality as many of the tools discussed above, but still serve an important purpose in designing experimental stimuli. WordGen is one such example, in which users can specify linguistic constraints to generate nonce words in Dutch, English, French, or German (Duyck et al., 2004). The main downside to WordGen is that it is a standalone Windows program and cannot be easily integrated into a broader programming workflow. Wuggy is a similar tool for word generation that takes the utility of WordGen a step further. It supports more languages, including Spanish and Vietnamese, and has a Python library (Keuleers & Brysbaert, 2010).

## The UCI Phonotactic Calculator

The UCI Phonotactic Calculator (henceforth UCIPC; https://phonotactics.socsci.uci.edu/) is an online tool we have developed that can be used to calculate various metrics to quantify phonotactic information. This tool has several primary differences from the existing tools discussed above:

1. It allows the user to specify their own training dataset. To our knowledge, this is the only online tool supporting this functionality for phonotactic metrics (note that CLEARPOND does support this, but for neighborhood density alone). This allows the UCIPC to be deployed on any language or input register (e.g., infant-directed speech).
2. It supports a wider range of metrics than most existing tools.
3. It can be run both via an online interface and via the command line, which allows it to be integrated into larger programming workflows.
4. It is open source: https://github.com/connormayer/uci_phonotactic_calculator.

Training and test data files uploaded by the user are stored on a secure server. Files that are more than 10 min old are deleted by an automated process that runs every 10 min, meaning that, in the worst case, the longest a file will persist on the server is about 20 min. If data privacy is a major concern, the user can download the UCIPC source code from the GitHub repository and calculate the metrics locally, without the data ever leaving their computer (see Appendix A).

### Currently supported phonotactic metrics

The UCIPC supports a range of phonotactic metrics that differ in how or whether they encode context, token frequency, and position. This section will provide a qualitative and quantitative description of each metric. The set of metrics is roughly divided into four classes based on the following two factors:

- *Unigram vs. bigram metrics*: do we consider the preceding context in which a sound occurs (bigram) or not (unigram)?
- *Absolute positional vs. relative positional metrics*: do we consider the absolute position in the word in which a unigram or bigram occurs or not? The absolute positional metrics correspond to the calculations done in Jusczyk et al. (1994) and Vitevitch and Luce (2004), while the relative positional metrics correspond to more standard implementations of n-gram models with word boundary symbols (Bahl et al., 1983; Chen & Goodman, 1999; Jurafsky & Martin, 2025; Markov, 1913; Shannon, 1948).

#### Relative positional unigram probabilities

In all of the sections below, we will use $w = {x}_{1}\dots {x}_{n}$ to refer to a word consisting of symbols ${x}_{1}$ through ${x}_{n}$.

The relative positional unigram score reflects the probability of a word under a standard unigram model. Here, the probability of a word is defined as the product of the probabilities of its individual symbols. In the relative positional version of unigram metrics, probabilities are calculated without considering the position of symbols. Rather, only the frequencies of symbols are used in the calculation. If a particular symbol exists in the test dataset but not in the training set, its probability is set to zero. Mathematically, we express the relative positional unigram probability of a word as

$$P\left(w={x}_{1}\dots {x}_{n}\right)\approx \prod \limits_{i=1}^{n}P\left({x}_{i}\right)$$

where we express the probability of encountering an individual unigram as

$$P\left(x\right)=\frac{C\left(x\right)}{{\sum }_{y\in\Sigma }C\left(y\right)}$$

where $C\left(x\right)$ represents the number of occurrences of $x$ in the training data and $\Sigma$ is the set of all sounds in the training data. $C\left(x\right)$ is divided by the total count of every symbol $y\in\Sigma$ in the training data, meaning that unigram probabilities are simply the relative frequency of the sound $x$. The UCIPC returns log unigram probabilities. It is important to note that the training process for all the models in this paper is deterministic: the unigram/bigram probabilities learned by the model will always be the same for a given input.

#### Absolute positional unigram scores

The UCIPC also computes absolute positional unigram scores, following the approach in Vitevitch and Luce (2004). These differ from the relative positional unigram probabilities above in that they are sensitive to the absolute position of each segment in a word, as well as its identity. The absolute positional unigram score is a type-weighted variant of the unigram score from Vitevitch and Luce (2004). It is defined as follows:

$$PosUniScore\left(w={x}_{1}\dots {x}_{n}\right) = 1 + \sum \limits_{i=1}^{n}P\left({w}_{i}={x}_{i}\right)$$

where

$$P\left({w}_{i}=x\right) = \frac{C\left({w}_{i}=x\right)}{{\sum }_{y \in\Sigma }C\left({w}_{i}=y\right)}$$

where $w_i$ refers to the *i*th position in a word and $C\left({w}_{i}=x\right)$ is the number of times in the training data the symbol *x* occurs in the *i*th position of a word.

Vitevitch and Luce (2004) add 1 to the sum of the unigram probabilities “to aid in locating these values when you cut and paste the output […] to another program” (p. 484). They recommend subtracting 1 from these values before reporting them.

Under this metric, the score assigned to a word is the sum of the probability of its individual symbols occurring at their respective positions. Higher scores represent words with more probable segments.

It is important to keep in mind that the values the absolute positional models associate with word forms are not valid probabilities, because the probabilities of individual unigrams and bigrams are combined by addition rather than multiplication. However, even if multiplication were used, this could not be interpreted as a probability distribution over words, since the probability of a sequence is generally not equal to the product of its joint bigram probabilities. The relative positional models, by comparison, decompose the probability of a sequence into the product of conditional probabilities using the chain rule of probability, and then approximate the individual conditional probabilities using the Markov assumption (see Jurafsky & Martin, 2025, Ch. 3). Therefore, users need to be aware that the behavior of absolute positional metrics is not mathematically well defined, and is thus more difficult to predict and reason about.

There is also an additional practical difference between absolute and relative positional models that emerges from the use of addition in the former models: all else being equal, longer words will have higher scores under the absolute model because the individual n-gram probabilities are combined by addition, and thus each additional symbol increases the score, favoring longer words. In the relative model, on the other hand, shorter words are preferred, because the individual n-gram probabilities are combined by multiplication, and thus each additional symbol decreases the score. A preference for longer words runs counter to a bias towards shorter words that is often employed in phonotactic modeling (e.g., Goldwater et al., 2009; Johnson et al., 2015; see also Storkel, 2004, for versions of the absolute model that are not sensitive to word length).

#### Relative positional bigram probabilities

Unlike unigram metrics, the bigram metrics consider pairs of symbols instead of individual symbols. Calculating the relative positional bigram score is done in a similar way to that of the relative positional unigram scores. That is, it is represented as the product of probabilities of consecutive symbols in each word conditioned on the previous symbol. Like the relative positional unigram model, the absolute position of the bigrams in the word is not considered, and bigrams not occurring in the training data are assigned a probability of zero. To incorporate information about word boundaries, we pad the words with a special symbol at the beginning and end, which allows us to compute the probabilities of symbols starting and ending words. For example, the input /kæt/ “cat” would consist of the bigrams {#k, kæ, æt, t#}, where # is a word boundary symbol. This allows the model to be sensitive to the frequencies with which certain segments begin and end words.

Following the standard definition of a bigram model (Jurafsky & Martin, 2025, Ch. 3), we define the bigram probability of a word as

$$P\left(w={x}_{1}\dots {x}_{n}\right)\approx \prod \limits_{i=2}^{n}P\left({x}_{i}|{x}_{i-1}\right)$$

where the probability of a particular bigram is calculated as

$$P\left(x|y\right)=\frac{C\left(yx\right)}{C\left(y\right)}$$

where the count function $C\left(\bullet \right)$ is defined as in Sect. "Relative positional unigram probabilities". The UCIPC returns log bigram probabilities.

#### Absolute positional bigram metrics

The UCIPC also computes absolute positional bigram scores. This is a type-weighted variant of the bigram score from Vitevitch and Luce (2004). It is defined as

$$PosBiScore\left(w={x}_{1}\dots {x}_{n}\right) = 1 + \sum \limits_{i=2}^{n}P\left({w}_{i-1}={x}_{i-1}, {w}_{i}={x}_{i}\right)$$

where

$$P\left({w}_{i-1}={x}_{i-1}, {w}_{i}={x}_{i}\right) = \frac{C\left({w}_{i-1}={x}_{i-1}, {w}_{i}={x}_{i}\right)}{{\sum }_{z \in\Sigma }{\sum }_{v \in\Sigma }C\left({w}_{i-1}=z, {w}_{i}=v\right)}$$

The same caveats apply here with respect to these scores not forming valid probabilities. These scores also differ from relative positional bigrams in that the bigram probabilities used to calculate the overall scores are *joint* probabilities rather than conditional probabilities: they tell us the probability of segment $x$ occurring in position $i$ *and* segment $y$ occurring in position $i+1$, while the relative positional bigram probabilities tell us the probability of segment $y$ occurring in position $i+1$ *given that* segment $x$ occurred in position $i$.

#### Token frequency-weighted metrics

In the standard metrics, which we call type-weighted, the frequency of individual word types does not affect the output scores. That is, word types that occur more frequently are weighted the same as word types that occur very few times. The token-weighted variants of each metric do account for frequency of word types. Specifically, each occurrence of a particular configuration is weighted by the natural log of the count of the word it occurs in.

For example, consider a corpus that contains the word type /kæt/ 1,000 times and /tæk/ 50 times. Under a token-weighted unigram model, we would have $C\left(t\right)=In\left(1,000\right)+In\left(50\right)\approx10.82$, whereas in a type-weighted unigram model, we would have $C\left(t\right)=1+1=2$.

The token-weighted absolute positional unigram and bigram scores correspond to the metrics in the PPC (Vitevitch & Luce, 2004). Although both the PPC and the UCIPC use log frequency counts, the PPC uses the base 10 logarithm, while the UCIPC uses the natural logarithm (logarithm with base $e$). Because logarithms with different bases differ only in a constant multiplicative factor, the choice of base does not have an impact on the relative differences between word scores.

#### Smoothed metrics

The calculator also calculates variants of every metric with add-one smoothing (Jeffreys, 1948, Sect. 3.23). With this type of smoothing, every n-gram has a default count of 1. Thus, n-grams that are not encountered in the training set, but do appear in the test set, are treated as if they occurred once in the former rather than not at all. This assigns a small probability to such configurations. Without smoothing, the count for an unencountered n-gram will be 0, and hence the model will assign it a probability of 0. The effect of zero-probability n-grams on word scores differs between the relative and absolute positional models. In the relative positional models, where word scores are computed by taking the product of the individual n-gram probabilities, this means that any word in the training data with an unattested n-gram will be assigned a probability of zero, making it indistinguishable from other words containing unattested n-grams, even if the probabilities of other n-grams in the words differ substantially. The effect of smoothing on the absolute positional models is less dramatic because probabilities are combined using addition. In unsmoothed models, unattested sequences have no effect on word scores, while in smoothed models, they will result in a small increase. Performing smoothing in the token-weighted versions of metrics is done by simply adding 1 to the log-weighted counts.

#### Summary of UCIPC metrics

To summarize, the UCIPC can compute the following metrics given training and test data:

1. Relative positional unigram probability
2. Relative positional bigram probability
3. Smoothed relative positional unigram probability
4. Smoothed relative positional bigram probability
5. Frequency-weighted relative positional unigram probability
6. Frequency-weighted relative positional bigram probability
7. Smoothed, frequency-weighted relative positional unigram probability
8. Smoothed, frequency-weighted relative positional bigram probability
9. Absolute positional unigram score
10. Absolute positional bigram score
11. Smoothed absolute positional unigram score
12. Smoothed absolute positional bigram score
13. Frequency-weighted absolute positional unigram score
14. Frequency-weighted absolute positional bigram score
15. Smoothed, frequency-weighted absolute positional unigram score
16. Smoothed, frequency-weighted absolute positional bigram score

All metrics except the absolute positional variants are reported as log probabilities.

### A brief tutorial for the UCIPC

The UCIPC requires two inputs: a training file and a test file. For the training set, users have the choice of uploading their own file or selecting from the several existing datasets readily available to the UCIPC. To choose an existing dataset, users may use the dropdown menu, which contains a short description of the available datasets. For a more detailed description of each file, users should view the Datasets page, which is dedicated to storing and explaining the use case for each dataset. Existing datasets include English, Spanish, Turkish, and Polish corpora (referenced below in this paper) as well as the Finnish, French, and Samoan datasets used in Mayer (2020).

If uploading a personal training file, users must take care to follow a few specifications:

- The file must be in CSV format.
- The file must consist of one or two columns without headers.

The first column is mandatory and should consist of a word list where symbols (phonemes, phones, letters, etc.; see Sect. "Application to domains beyond phonotactics") are separated by spaces. Any transcription system is valid, so long as individual symbols are space-separated. The second column is optional and, if included, should contain the corresponding frequencies for each word, expressed as counts. If this column is included in the training file, both the type- and token-weighted variants of each metric will be computed. Otherwise, just the type-weighted metrics will have values in the output file, and the token-weighted metrics will have NaN values. Note that users may not both upload their own training file *and* select a default training file; the UCIPC will display an error message requesting a single choice to be made.

The test file needs to be a CSV file with a single column of test words and no headers. The transcription system used in the test file should match the system used in the training file. The mechanism for uploading the test file is the same as uploading the training file. It is generally the case that test files will contain data not found in the training set in order to test the models’ ability to generalize and avoid the risk of overfitting to the training data (e.g., Ying, 2019), but this is not required. A test file that partially or completely overlaps with the training forms can also be used if the intention is simply to calculate scores for particular lexical items, rather than to evaluate the capacity of the models to generalize.

Once users submit their training file, test file, and model type, the UCIPC will direct them to a separate page to download the output file. Users will receive a CSV file where each row contains the test word, its length, and all the calculated variations of the unigram and bigram metrics. To run the model again, users must go back to the UCIPC home page and resubmit the input form with the necessary fields (training/test file, model type). Because both files uploaded to the server and the output CSV files are cleaned frequently, users should be sure to download their output data within 10 min of generating it.

## Applications of phonotactic metrics in experiments with adults

In this section, we model phonotactic acceptability ratings given by human participants in published studies as a function of a variety of phonotactic metrics. Our purpose here was to determine whether metrics that encode absolute positional information or relative positional metrics that take word edges but no other positional information into account are best able to predict acceptability judgments by adult native listeners. For each of the following datasets, we run the UCIPC with an appropriate training set in the same language. The calculator’s outcomes are used as predictors in a regression model that attempts to predict the human ratings. All the code and data can be found at https://github.com/aryarksub/phonotactic_metrics.

The general format for each regression model is

$$Acceptability \sim UnigramScore*BigramScore$$

with random intercepts included for individual participants and items when the data were sufficiently granular. Because the model includes an interaction term, the unigram and bigram score predictors are mean-centered, by subtracting the mean from each observation, and scaled to *Z*-scores, by dividing each centered observation by the standard deviation. Outputs from the UCIPC that are negative infinity (corresponding to a probability of zero) are adjusted to a large negative value (e.g., − 50) so that scaling can be done without error. The specific type of regression used (linear or logistic) depends on the experimental design of each study.

We consider eight models for each dataset resulting from the combination of type of metric (relative positional or absolute positional), whether or not metrics were smoothed, and whether or not the metrics were token frequency-weighted. Token-weighted metrics are omitted when frequency information is not available for the training data.

We compare the performance of models using the Akaike information criterion (AIC; Akaike, 1974). The AIC is a metric for model comparison that estimates out of sample prediction error. It rewards model fit to the data and penalizes model complexity. Lower values of AIC indicate better model performance. However, absolute AIC values are not meaningful, but differences in AIC between a model and the model with the lowest AIC can be used to evaluate their performance on a dataset. We interpret differences in the AIC using the rule of thumb proposed in Burnham and Anderson (2004; p. 271): an AIC difference of ≤ 2 between a model $M$ and the model with the lowest AIC score ${M}_{min}$ means there is “considerable support” for $M$(i.e., $M$ and ${M}_{min}$ are both plausibly the best model); a difference of between 4 and 7 means $M$ has “considerably less support” relative to ${M}_{min}$, and a difference of more than 10 indicates “essentially no support” for $M$ relative to ${M}_{min}$.

For each of the four languages, we briefly summarize each study whose data we use and then present the results for all datasets in a single table. The summary table displays the corresponding AIC for each combination of model and dataset. The best-performing model on each dataset (the model with the lowest AIC) is highlighted in bold.

### English

Unsurprisingly, the greatest number of reports are on native English speakers’ phonotactic judgments, as is the case in psycholinguistics more generally (Vitevitch et al., 2014; Blasi et al., 2022). In this section, we report results from modeling data obtained from five published studies.

#### Albright and Hayes (2003)

The data used in Albright and Hayes (2003) comprise 58 English monosyllabic nonce verbs consisting of between three and five segments rated for phonological well-formedness by 20 native English speakers. All stimuli were presented auditorily. These data correspond to the pretest portion of Experiment 1 in their paper. Participants were asked to rate forms on a Likert scale between 1 (impossible as an English word) and 7 (would be a fine English word). The data are in the form of mean ratings across participants; individual ratings are not available.

The phonotactic models were trained on the English CMU Pronouncing Dictionary (CMU Pronouncing Dictionary, 2008) with frequency information from CELEX (Baayen et al., 1995). Stress location was not represented in the training data. The fitted models provided scores for the 58 nonce verbs used in the study. We used these scores as predictors in a set of linear models to model the mean rating. Because the dataset does not contain individual ratings by subject, we do not use any random effects.

#### Daland et al. (2011)

The test data obtained from Daland et al. (2011) consist of 96 disyllabic English nonce words, each six segments long. These nonce words were rated on a five-point Likert rating scale by 48 native English speakers and the ratings were aggregated across participants. All stimuli were presented orthographically. The main goal of Daland et al. (2011) was to compare the acceptability of different onsets in English. These nonce words accordingly consist of a set of 48 complex onsets (e.g., /tw/, /vr/, /bl/, etc.) and six “tails” to complete the word (e.g., /-ɑtɪf/, /-ɛzɪg/). Each onset was paired with two of the six tails, resulting in a total of 96 nonce words. Models were fit to the same English training dataset as described in the previous section, and the 96 nonce words (including tails) were scored by the fitted models. We used these scores as predictors in a linear regression model that uses the mean word ratings across participants as its output feature. Because scores were aggregated across participants, there were no random effects in the model.

#### Needle et al. (2022)

The data from Needle et al. (2022) consist of ratings of 8,400 English nonce words by 1,440 participants. Nonce words consisted of 4–7 segments. All stimuli were presented orthographically. Each participant rated 140 stimuli each, leading to 24 ratings for each individual nonce word. Ratings were provided on a five-point Likert scale.

In this case, the training dataset we use is the same as that used in Needle et al. (2022) and consists of about 11,000 monomorphemic words from CELEX (Baayen et al., 1995) in the DISC transcription system. We converted the DISC transcriptions to ARPABET to stay consistent with the system used for English throughout this paper. Because these training data do not contain frequency information, the token-weighted models could not be used. The other models were fitted to these training data and used to score the experimental stimuli. These scores were used as predictors in a linear mixed-effects model, with random intercepts for word and participant.

#### Scholes (1966)

The test data from Scholes (1966) were obtained from the supplementary material of Hayes and Wilson (2008). It consists of 62 monosyllabic nonce words rated by 33 seventh-grade students. Words were presented orthographically. These words varied primarily in their onsets. The students were asked whether each word was a possible word in English and asked to provide a yes/no response; thus, the data here consist of binary responses rather than Likert scores. The data were aggregated across onset, which means each of the 62 onsets is associated with a value between 0 and 1 that represents the proportion of “yes” responses across participants.

The training data used were also from the supplementary materials of Hayes and Wilson (2008) and consist of 55 English onsets and their type frequencies. This is a subset of the onsets in the CMU Pronouncing Dictionary with “exotic” onsets like /zw/ and /sf/ removed. This is rather different from the training datasets in previous cases, because our training data consist of onsets, rather than words, and our frequency counts correspond to the number of word types each onset occurs in. The models were trained on this dataset and tested on the 62 words from Scholes (1966); these testing data also come from the supplementary material for Hayes and Wilson (2008). The model scores were used as predictors in a logistic regression model over the proportions, weighted by the number of participants. Because we do not have individual ratings, we do not include any random effects.

#### Hayes and White (2013)

The test data procured by Hayes and White (2013) consist of 160 English nonce words consisting of between two and seven segments rated on a logarithmic scale by 29 participants. Stimuli were presented simultaneously in both orthographic and auditory form. Participants were asked to perform a magnitude estimation task (Bard et al., 1996; Lodge, 1981) comparing the well-formedness of each word with the reference word “poik.” The log of these magnitudes is the dependent variable we use here. The training data are the same CMU Pronouncing Dictionary data used in the analyses of Albright and Hayes (2003) and Daland et al. (2011). Models were trained on these data and used to score each nonce word. These scores were used as predictors in a linear mixed-effects model with random intercepts for participant and word.

#### English results

Table 1 shows the AIC of each of the eight model types on the five English datasets. These results show that the best-performing model in each case is a relative positional model. Indeed, relative positional models almost always outperformed their absolute positional counterparts, the sole exception being the data from Daland et al. (2011), where some absolute positional models outperformed their relative positional counterparts (though in this case the best-performing model is still a relative positional one). The differences in AIC between the best relative positional model and best absolute positional model are > 10 in all cases, indicating strong support for the relative positional models; the exception is Scholes (1966), where the difference is 2.16, indicating weak support for the relative positional models. Smoothing generally results in a decreased AIC, except on the Needle et al. (2022) data. The non-frequency-weighted models generally perform better than the frequency-weighted ones, though this is not the case for the Scholes (1966) and Hayes and White (2013) data (this difference is minor in the former case). We will discuss this phenomenon more in Sect. "Other languages" when we look at Polish onsets, where the effect is much stronger.

**Table 1:** AIC scores of the regression models fit to the data of all five published studies in English; full model results with coefficients are available in Appendix B. The best-performing model in each column is highlighted in bold

Model	Albright and Hayes (2003)	Daland et al. (2011)	Needle et al. (2022)	Scholes (1966)	Hayes and White (2013)
Relative positional	123.965	284.538	**566,148.8**	36.65767	12,507.21
Relative positional + smoothed	**123.115**	**242.270**	566,288.5	36.03306	12,349.81
Relative positional + frequency-weighted	124.152	284.260	–	36.53359	12,519.93
Relative positional + frequency-weighted + smoothed	123.437	244.621	–	**35.40623**	**12,338.82**
Absolute positional	129.706	260.176	570,030.7	39.76660	13,013.83
Absolute positional + smoothed	129.714	259.450	570,084.3	41.47684	13,014.93
Absolute positional + frequency-weighted	129.562	258.719	–	38.09191	13,009.03
Absolute positional + frequency-weighted + smoothed	129.566	258.460	–	37.56650	13,009.36

### Other languages

The remaining three studies we discuss are on non-English languages. We describe them together in this section.

#### Polish (Jarosz & Rysling, 2017)

The Polish test data we use come from Jarosz and Rysling (2017). In this paper, 81 native Polish speakers were asked to rate 159 test words consisting of 53 onsets and three tails (similar to the design in Daland et al., 2011) on a Likert scale of 1–5. Each participant rated each word once, leading to 12,880 responses. Stimuli were presented orthographically.

Our training dataset consisted of the list of Polish onsets with accompanying type frequencies from Jarosz (2017). These are generated from a corpus of child-directed speech consisting of about 43,000 word types (Haman et al., 2011). Because we trained only on onsets, we generated model predictions for the 53 onsets in isolation (meaning that the three tails corresponding to each onset receive the same score). The model scores are used as predictors in a linear mixed-effects model with random intercepts for word (including tail) and participant.

#### Spanish

This dataset was collected by authors CM and MS using the methodology from Sundara and Breiss (under review) for use in an unrelated study that is still in progress (Mayer & Sundara, in prep). The data consist of 576 unique consonant–vowel-consonant–vowel (CVCV) Spanish nonce words rated on a discrete scale from 1 to 100 by 168 participants. Each participant rated 144 tokens, leading to 24,192 ratings. Stimuli were presented simultaneously in both orthographic and auditory form. The phonotactic models were trained on a set of about 27,000 word types including citation and inflected forms taken from the EsPal database (Duchon et al., 2013) with stress encoded. The frequencies associated with these words were calculated from a large collection of Spanish subtitle data. The trained models were used to score the 576 nonce words. We use these scores as predictors in a linear mixed-effects model with random intercepts for participants and words. Random intercepts are used for individual words and subjects.

#### Turkish

The test data, described in more detail in Mayer (2024, 2025), consist of 596 Turkish consonant–vowel-consonant–vowel-consonant (CVCVC) nonce words rated on a discrete scale from 1 to 100 by 90 participants following the same methodology as the Spanish study above. Each participant rated 192 tokens, leading to 17,280 ratings. Stimuli were presented simultaneously in both orthographic and auditory form. The phonotactic models were trained on a set of 18,472 citation forms from the Turkish Electronic Living Lexicon database (TELL; Inkelas et al., 2000). These training data do not contain frequency information, so we omit results from the frequency-weighted models. Fitted models were used to generate scores for the 596 nonce words. These scores were used as predictors in a linear mixed-effects model with random intercepts for word and participant.

#### Other language results

Table 2 shows again that the best models are generally the relative positional, smoothed metrics without frequency weighting. The difference in AIC between the best-performing relative positional model and the best-performing absolute positional model on each dataset was > 10, indicating strong support for the relative positional models. Although frequency weighting was generally not beneficial, the Polish dataset from Jarosz and Rysling (2017) was an exception. Similar to the data from Scholes (1966) presented above, but more pronounced, frequency weighting appears to be crucial for model performance. This may reflect some language-specific sensitivity to frequency. However, as with the Scholes (1966) data, the training data consist of onsets with type frequencies rather than words with token frequencies. When a non-frequency-weighted model is applied to these data, the training data consist of a simple list of attested onsets lacking both type and token frequency information. It is more likely, therefore, that the success of the frequency-weighted models here corresponds to a sensitivity to type frequency information. It is less clear why the Hayes and White (2013) English data benefit from frequency weighting.

**Table 2:** AIC scores of the regression models fit to the data of studies on Polish, Spanish, and Turkish; full model results with coefficients are available in Appendix B. The best-performing model in each column is highlighted in bold

Model	Jarosz & Riesling (2017) Polish	Mayer & Sundara (in prep) Spanish	Mayer (2024, in press) Turkish
Relative positional	44,883.97	187,932.9	159,581.9
Relative positional + smoothed	44,799.76	**187,729.1**	**159,545.6**
Relative positional + frequency-weighted	44,849.67	188,059.9	–
Relative positional + frequency-weighted, + smoothed	**44,609.70**	188,059.9	–
Absolute positional	44,908.04	189,100.6	159,628.4
Absolute positional + smoothed	44,907.11	188,252.1	159,628.8
Absolute positional + frequency-weighted	44,836.69	189,668.1	–
Absolute positional, + frequency-weighted + smoothed	44,835.34	189,668.3	–

### Discussion

Several clear trends emerge from the results presented above. Relative positional models outperform positional models in every case. Relative positional smoothed models generally outperform their unsmoothed counterparts. However, for absolute positional models, smoothing typically has little effect and sometimes reduces their performance: this is not unexpected given the discussion in Sect. "Smoothed metrics". Finally, frequency-weighted models generally perform similarly to or slightly worse than non-frequency-weighted models. The exceptions to this are Hayes and White (2013), Scholes (1966), and Jarosz and Riesling (2017). The second and third cases are not really exceptions, because the frequency information in the training data consists of onset type frequencies rather than token frequency: the success of the frequency-weighted models in these cases simply indicates that type frequency is important. The only true exception is Hayes and White (2013), where including token frequency improved the performance of the smoothed model.

What is even more striking about these results is that they emerge across a range of different domains and languages: some studies, like Scholes (1966), Daland et al. (2011), and Jarosz and Rysling (2017), focus only on onsets, while the others look at whole word forms. In some, the stimuli are presented orthographically, in others auditorily, and in others, both. In all cases, relative positional models that encode information about word edges, but no other absolute position information, best predict native speaker judgments.

Why should it be the case that relative positional models do better? There are several possible reasons. First, as mentioned earlier in Sect. "The Phonotactic Probability Calculator", absolute positional models have issues with data sparsity which make it difficult for them to assign accurate scores to long words: estimates for later positions will necessarily be based on less data than for earlier positions, because there are fewer words with material in those positions. Thus, we might expect scores assigned to longer words to be less useful in predicting human behavior. However, the maximum length of test words in the eight studies we looked at was seven segments, and even in published results on monosyllabic nonce words with fewer segments or onsets alone (Albright & Hayes, 2003; Scholes, 1966; Jarosz & Riesling, 2017), relative positional models outperformed absolute positional ones.

Second, the relative positional metrics can capture phonotactic constraints that target both word-initial and word-final material, which are often important positions in terms of phonotactic constraints (e.g., Beckman, 1997; Lombardi, 1999) and an important source of information in word segmentation and allophonic learning for adults (e.g., Endress et al., 2009; Newman et al., 2011; Skoruppa et al., 2015) and for infants (e.g., Jusczyk et al., 1999; Katsuda & Sundara, 2024). In native Turkish words, for example, /ɾ/ can never begin a word, and words cannot end in voiced stops or affricates. The relative positional models can encode these restrictions with the use of boundary symbols: such a model trained on Turkish would assign a low probability to the sequences /#ɾ/ and /b#/, where # is a word boundary, reflecting the prohibition on word-initial /ɾ/ and word-final voiced stops. Although the absolute positional models can encode word-initial constraints, since every occurrence of a sound in position 1 is a word-initial occurrence, they cannot encode word-final constraints, as the position of the final element in a word depends on its length. An absolute positional model struggles to encode restrictions like Turkish’s ban on final voiced stops: a /b/ in the third position could have a high probability if a word is of length five (as in /babam/ “my father”), but a low probability if it is of length three.

Finally, in addition to these factors, it may simply be the case that relative positional models correspond better to human cognitive processes than absolute positional ones do, because humans do not take absolute position into account, because they compute conditional rather than joint probabilities, because they are biased to prefer shorter words (e.g., Goldwater et al., 2009; Johnson et al., 2015), or some combination of these. A more detailed investigation using tools such as the UCIPC will be useful in teasing these factors apart.

These results have important implications. First, absolute positional metrics of phonotactics, at least as currently implemented, do not predict human phonotactic generalization as well as relative positional metrics: the relative positional metrics outperform the absolute positional ones in every case. This suggests that the absolute positional metrics used by many phonotactic calculators, including the popular Phonotactic Probability Calculator (Vitevitch & Luce, 2004), may not be the most suitable for modeling human acceptability judgments.

Second, it is generally the case that models that take token frequency into account perform more poorly than models that do not: this is somewhat less clear cut, however. The greater utility of type (vs. token) frequency to model human behavior has also been reported in domains besides phonotactic judgments (Albright, 2002; Albright & Hayes, 2003; Bybee, 1995, 2003; Goldwater, 2007; Hayes & Londe, 2006; Hayes & Wilson, 2008; Pierrehumbert, 2001; Richtsmeier, 2011). Further research is needed to determine the circumstances under which speakers are sensitive to token frequency when forming phonotactic judgments, and in language processing more generally (e.g., Conrad et al., 2008; del Prado Martin et al., 2004; Ellis, 2002; Endress & Hauser, 2011).

Finally, smoothed models generally outperform unsmoothed models. This is not surprising: speakers do not judge words containing unattested sequences as totally unacceptable. It is important to note, however, that the add-one smoothing used in these models is rather coarse, assigning each unattested sequence the same pseudo-count. It has been well established in linguistic research that speakers generalize to unattested sequences based on their similarity to existing sequences in the language (e.g., Chomsky & Halle, 1965, 1968; Hayes & Wilson, 2008; Wilson & Gallagher, 2018; Dai et al., 2023, a.o.). More robust smoothing metrics that can capture these differences would be valuable but are beyond the scope of the current paper.

One important limitation of this research is that all of the data we consider here are phonotactic ratings. Castro and Vitevitch (2023) note that different phonotactic metrics may be more predictive depending on the task itself (e.g., reading time as opposed to acceptability judgments) and more specific details of the task such as the presence of noise, time pressure, and so on. Although these results support relative positional metrics as the best predictors of acceptability judgments, it remains to be seen whether this will hold across other tasks to which phonotactic sensitivity is relevant.

## Applications of phonotactic metrics in stimulus construction

In addition to serving as variables of interest in experimental work or computational models of speech, the metrics calculated by the UCIPC are also useful for constructing and selecting experimental stimuli. As shown in the previous sections, the UCIPC can be used to calculate a wide array of metrics to summarize how likely segments and segment sequences are in any given corpus. Such metrics are also extremely useful when constructing stimuli for experiments.

### Experiments with infants

The UCIPC can be used to quantify the extent to which some segments or segment sequences are frequent in any language for which a phonologically transcribed lexicon or corpus of speech is available. Such quantification is necessary when manipulating segment or segment sequence frequencies as an independent variable in experiments designed to determine when, if at all, infants are sensitive to native language patterns (e.g., Archer & Curtin, 2011; Friederici & Wessels, 1993; Gonzalez-Gomez & Nazzi, 2012). Quantification is also necessary to identify and index experimental confounds when differences in segment and segment sequence likelihood are not the target of inquiry but are nonetheless likely to influence infant behavior (Gonzalez-Gomez & Nazzi, 2012; Nazzi et al., 2009; Sebastián-Gallés & Bosch, 2002; Solá-Llonch & Sundara, 2025).

In addition to standardizing the calculations of metrics to promote replicability, tools like the UCIPC allow new investigators with more modest technical backgrounds, particularly those working on under-resourced languages, to employ phonotactic models in their research. Typically, to develop stimuli in a new language, an investigator would need access to a corpus, as well as computational skills to conduct corpus analyses to identify patterns and index the incidence of sounds and sound sequences. With the UCIPC, metrics can be obtained for any language as long as there is a dataset with all the words in a dictionary or corpus listed in a consistent transcription system. With time, we expect to increase the number of pre-existing datasets for different languages, to alleviate the challenge of identifying suitably sized corpora in different languages.

### Experiments with artificial languages

The outcome of artificial language experiments has been reported to differ in adults with different native languages (e.g., Do & Yeung, 2021; Huang & Do, 2021; White et al., 2018). This is typically dealt with by either recruiting only participants who speak the same language(s), so that the same L1 biases are shared across participants, or using language background as a control variable in the analysis. Phonotactic metrics such as those generated by the UCIPC can also be useful in designing or analyzing artificial language learning experiments. For example, if a study were to be run on both English and Spanish speakers, phonotactic models fit to English and Spanish training data could be useful to score each stimulus and identify and remove cases where the models’ scores deviate substantially between languages. Alternatively, these scores could themselves be used as control variables, rather than the coarser metric of language background. This approach has the potential not only to better control for L1 effects in AGL, but also to quantify and predict them.

Finally, the artificial language itself can be used as the training data to ensure that the test items do not vary on segment and segment sequence likelihood that are themselves not the target of inquiry.

### Application to domains beyond phonotactics

Although the UCIPC is intended to be used as a model of phonotactics, it has applications in other domains as well. One clear application is in the study of orthotactics, which deals with restrictions on how orthographic symbols can be combined into words in a language, and how awareness of these restrictions influences tasks such as reading and spelling (e.g., Apel et al., 2006; Krasa & Bell, 2021). Computing orthotactic probabilities using the UCIPC is as simple as substituting orthographic symbols for phonetic symbols. Similarly, the UCIPC could also be deployed on morpheme sequences to compute morphotactic probability (e.g., Sproat, 1992; Crysmann & Bonami, 2016). Although the metrics computed by the UCIPC are unlikely to be useful for syntax, where it is common to have dependencies between non-adjacent words, morphological dependencies tend to be local in the same way as phonotactic dependencies (Aksenova et al., 2016), making n-gram models a suitable choice in many cases.

## Planned extensions of the UCIPC

Currently, the UCIPC does not implement calculation of neighborhood density. This is largely due to the contexts in which it has been applied so far: we have focused primarily on phonotactic acquisition in the first year, and previous research has indicated that infants are not sensitive to neighborhood density during this time period (Sundara et al., 2022; Swingley & Aslin, 2002). To make the UCIPC more applicable to the study of adult phonotactic knowledge, we plan to implement this functionality soon. Although there are online tools that support neighborhood density measurements in a wide range of languages (e.g., Alzahrani, 2025, as well as some discussed in Sect. "Limitations of existing tools for computing phonotactic acceptability" above), the UCIPC could provide greater flexibility by allowing neighborhood density measurements to be computed for arbitrary training data.

We also plan to add more sophisticated smoothing techniques. Currently, all smoothed metrics involve add-one smoothing. This technique has the virtue of being simple, but it tends to shift too much probability mass from observed to unobserved word forms. We plan to add additional smoothing techniques, such as modified Kneser–Ney or Witten–Bell smoothing, which have been shown to perform more favorably in NLP tasks (e.g., Chen & Goodman, 1999). To our knowledge, no work has looked at smoothing as it relates to modeling phonotactic acceptability judgments. A more detailed study of how well different smoothing techniques correlate with empirical observations in this domain will be valuable.

Finally, we would like to emphasize that the UCIPC is an open-source project (the source code can be found at https://github.com/connormayer/uci_phonotactic_calculator). If you are interested in adding new functionality or fixing bugs, please reach out to the corresponding author.

## Conclusion

In this paper we have presented the UCI Phonotactic Calculator, a new online tool that allows users to compute a suite of different phonotactic acceptability metrics. Compared to existing tools, the UCIPC has several desirable properties:

- Users can provide their own training data, allowing it to be applied to any language, whether natural or artificial, for which suitable data are available.
- It computes a large suite of different types of acceptability metrics.
- It has a simple point-and-click interface that allows it to be used by researchers with limited technical backgrounds.

Sect. "Applications of phonotactic metrics in experiments with adults" provided an example of how the calculator can be applied to answer questions about what aspects of phonotactic patterns speakers encode and how they generalize to unattested patterns. This demonstrated that, overall, models that are not sensitive to absolute position in the word or to token frequency do the best at predicting human judgments across a range of studies in four different languages.

The UCIPC has several valuable research applications in addition to modeling phonotactic acceptability. It can be used in stimulus construction for lexical decision tasks, infant experiments, or artificial grammar learning studies to control for the effects of phonotactic probability in participants’ native languages. It can also be used be used to model changes in phonotactic generalizations resulting from different hypotheses about infants’ changing lexicons (see, e.g., Sundara, Breiss, Dickson, & Mayer, under revision).

We hope that the UCIPC will be a valuable tool for researchers who are interested in phonotactic acceptability. We would like to close by emphasizing again that the UCIPC is an open-source project: the source code can be freely examined, and we welcome contributions from researchers who would like to add additional functionality or fix existing bugs.

## Supplementary Information

Below is the link to the electronic supplementary material.

Supplementary file1 (DOCX 28 KB)

## Data Availability

The UCI Phonotactic Calculator can be accessed at https://phonotactics.socsci.uci.edu/. The data and code used in the analyses in Sect. "Applications of phonotactic metrics in experiments with adults" can be found at https://github.com/aryarksub/phonotactic_metrics.

## Code availability

The code for the UCI Phonotactic Calculator can be found at https://github.com/connormayer/uci_phonotactic_calculator.

## Declarations

### Open practices statement

Links to the data and code are provided above in the Declarations section. The Turkish and Spanish studies were not preregistered.
