# Supplementary material for: The UCI Phonotactic Calculator: An online tool for computing phonotactic metrics
Source: Behav Res Methods. 2025 Jul 3;57(8):218. doi: 10.3758/s13428-025-02725-z (PMC12226677; doi:10.3758/s13428-025-02725-z)
Supplement: Supplementary file 1 — Supplementary file1 (DOCX 28 KB) [file 13428_2025_2725_MOESM1_ESM.docx]

**Appendix A**

Aside from the web interface, phonotactic metrics can also be calculated via the command-line interface for the UCIPC. To use the interface, users must download the UCIPC source code from the GitHub repository and, in their local terminal, navigate to the src directory which holds the ngram_calculator.py file. The calculator can then be run with the command

python ngram_calculator.py [train_file] [test_file] [results_file]

where the arguments refer to the local paths to the training file, test file, and output file to use, respectively. For example, using the command-line interface on sample files located in the data directory can be done as follows:

python ngram_calculator.py ..\data\english_cmu_freq.txt ..\data\sample_test_data\english_test_data.csv outfile.csv

**Appendix B**

Each of the following subsections examines an individual test dataset and reports the results of the relevant models as run on the corresponding data. The results are formatted in a tabular manner, with the following column headers:

- Model: Specifies the metrics used as predictors in the model
- Intercept: Regression intercept
- Uni. Coef: Coefficient for the unigram score term
- Bi. Coef: Coefficient for the bigram score term
- Int. Coef: Coefficient for the interaction (between unigram and bigram score) term
- AIC: Akaike Information Criterion (estimation of prediction error; Akaike 1974)

The models in each table are ordered by ascending AIC, with lower scores indicating better model performance.

**B.1** **English Models**

**B.1.1**  **Albright and Hayes (2003)**

| **Model** | **Intercept** | **Uni. Coef.** | **Bi. Coef.** | **Int. Coef.** | **AIC** |
| --- | --- | --- | --- | --- | --- |
| Relative Positional  + Smoothed | 4.69160 | 0.15713 | 0.11632 | -0.01857 | 123.115 |
| Relative Positional  + Frequency-weighted,  + Smoothed | 4.68971 | 0.16792 | 0.10045 | -0.01575 | 123.437 |
| Relative Positional | 4.70083 | 0.22337 | 0.07286 | -0.14096 | 123.965 |
| Relative Positional  + Frequency-weighted | 4.69640 | 0.22241 | 0.05458 | -0.11392 | 124.152 |
| Absolute Positional  + Frequency-weighted | 4.72162 | -0.11934 | 0.13720 | -0.05067 | 129.562 |
| Absolute Positional,  + Frequency-weighted  + Smoothed | 4.72111 | -0.11911 | 0.13690 | -0.05002 | 129.566 |
| Absolute Positional | 4.70156 | -0.10987 | 0.12717 | -0.02460 | 129.706 |
| Absolute Positional  + Smoothed | 4.70003 | -0.10882 | 0.12585 | -0.02265 | 129.714 |

**Table 3:** Coefficients and AIC scores of the regression models fit to data from Albright & Hayes (2003).

**B.1.2** **Daland et al. (2011)**

| **Model** | **Intercept** | **Uni. Coef.** | **Bi. Coef.** | **Int. Coef.** | **AIC** |
| --- | --- | --- | --- | --- | --- |
| Relative Positional  + Smoothed | 2.62606 | -0.08283 | 0.67846 | 0.29493 | 242.270 |
| Relative Positional  + Frequency-weighted,  + Smoothed | 2.61921 | -0.08328 | 0.68104 | 0.31075 | 244.621 |
| Absolute Positional,  + Frequency-weighted  + Smoothed | 2.75135 | -0.06492 | 0.66884 | -0.04975 | 258.460 |
| Absolute Positional  + Frequency-weighted | 2.74997 | -0.06262 | 0.66403 | -0.04779 | 258.719 |
| Absolute Positional  + Smoothed | 2.73739 | -0.03431 | 0.62606 | -0.03083 | 259.450 |
| Absolute Positional | 2.73305 | -0.02656 | 0.61117 | -0.02467 | 260.176 |
| Relative Positional  + Frequency-weighted | 2.62998 | -0.05006 | 0.42167 | 0.21011 | 284.260 |
| Relative Positional | 10.93989 | 0.34735 | 0.32775 | 0.01387 | 284.538 |

**Table 4:** Coefficients and AIC scores of the regression models fit to data from Daland et al. (2011).

**B.1.3** **Needle, Pierrehumbert & Hay (2022)**

| **Model** | **Intercept** | **Uni. Coef.** | **Bi. Coef.** | **Int. Coef.** | **AIC** |
| --- | --- | --- | --- | --- | --- |
| Relative Positional | 2.72212 | 0.05903 | 0.54662 | -0.00847 | 566178.8 |
| Relative Positional  + Smoothed | 2.66202 | -0.32281 | 0.69883 | 0.08722 | 566288.5 |
| Absolute Positional  + Smoothed | 2.79281 | -0.16563 | 0.33620 | -0.10043 | 570030.7 |
| Absolute Positional | 2.79435 | -0.15024 | 0.32053 | -0.10272 | 570084.3 |

**Table 5:** Coefficients and AIC scores of the regression models fit to data from Needle et al. (2022).

**B.1.4** **Scholes (1966)**

| **Model** | **Intercept** | **Uni. Coef.** | **Bi. Coef.** | **Int. Coef.** | **AIC** |
| --- | --- | --- | --- | --- | --- |
| Relative Positional  + Frequency-weighted,  + Smoothed | -0.58485 | 0.02450 | 1.93349 | 0.20597 | 35.40623 |
| Relative Positional  + Smoothed | -0.31853 | -1.12792 | 2.83191 | -0.24395 | 36.03306 |
| Relative Positional  + Frequency-weighted | -0.20491 | 0.55417 | 1.60020 | -0.30457 | 36.53359 |
| Relative Positional | -0.16237 | 0.51992 | 1.64383 | -0.36761 | 36.65767 |
| Absolute Positional,  + Frequency-weighted  + Smoothed | -0.11198 | 0.70349 | 1.65370 | -0.48528 | 37.56650 |
| Absolute Positional  + Frequency-weighted | -0.25016 | 0.84140 | 1.38754 | -0.21887 | 38.09191 |
| Absolute Positional | 0.70141 | 0.06254 | 3.19127 | -1.93961 | 39.76660 |
| Absolute Positional  + Smoothed | -0.00595 | 0.67075 | 2.32122 | -1.25656 | 41.47684 |

**Table 6:** Coefficients and AIC scores of the regression models fit to data from Scholes (1966).

**B.1.5** **Hayes and White (2013)**

| **Model** | **Intercept** | **Uni. Coef.** | **Bi. Coef.** | **Int. Coef.** | **AIC** |
| --- | --- | --- | --- | --- | --- |
| Relative Positional  + Frequency-weighted,  + Smoothed | 4.40106 | -0.35521 | 0.52082 | 0.02889 | 12338.82 |
| Relative Positional  + Smoothed | 4.39708 | -0.40281 | 0.55471 | 0.03242 | 12349.81 |
| Relative Positional | 4.41836 | -0.29086 | 0.44213 | 0.00310 | 12507.21 |
| Relative Positional  + Frequency-weighted | 4.41401 | -0.28490 | 0.43514 | 0.01021 | 12519.93 |
| Absolute Positional  + Frequency-weighted | 4.42823 | -0.02101 | 0.18375 | -0.00925 | 13009.03 |
| Absolute Positional,  + Frequency-weighted  + Smoothed | 4.42809 | -0.02090 | 0.18315 | -0.00907 | 13009.36 |
| Absolute Positional | 4.43072 | -0.03758 | 0.19762 | -0.01205 | 13013.83 |
| Absolute Positional  + Smoothed | 4.43027 | -0.03711 | 0.19561 | -0.01148 | 13014.93 |

**Table 7:** Coefficients and AIC scores of the regression models fit to data Hayes and White (2013).

**B.2** **Other languages**

**B.2.1** **Polish (Jarosz & Riesling 2017)**

| **Model** | **Intercept** | **Uni. Coef.** | **Bi. Coef.** | **Int. Coef.** | **AIC** |
| --- | --- | --- | --- | --- | --- |
| Relative Positional  + Frequency-weighted,  + Smoothed | 3.08772 | 0.00761 | 0.72491 | 0.07526 | 44609.70 |
| Relative Positional  + Smoothed | 3.09279 | -0.02533 | 0.68918 | 0.06116 | 44799.76 |
| Absolute Positional,  + Frequency-weighted  + Smoothed | 3.22977 | 0.30610 | 0.58109 | -0.19084 | 44835.34 |
| Absolute Positional  + Frequency-weighted | 3.22888 | 0.30468 | 0.58098 | -0.18967 | 44836.69 |
| Relative Positional  + Frequency-weighted | 3.05181 | 0.05792 | 0.63124 | 0.18117 | 44849.67 |
| Relative Positional | 3.05091 | -0.03312 | 0.67438 | 0.15339 | 44883.97 |
| Absolute Positional  + Smoothed | 3.14070 | 0.42246 | 0.34818 | -0.05221 | 44907.11 |
| Absolute Positional | 3.14046 | 0.42175 | 0.34839 | -0.05175 | 44908.04 |

**Table 8:** Coefficients and AIC scores of the regression models fit to data from Jarosz & Riesling (2017).

**B.2.2** **Spanish (Mayer and Sundara in prep)**

| **Model** | **Intercept** | **Uni. Coef.** | **Bi. Coef.** | **Int. Coef.** | **AIC** |
| --- | --- | --- | --- | --- | --- |
| Relative Positional  + Smoothed | 51.07835 | -1.03073 | 8.11025 | 1.32290 | 187729.1 |
| Relative Positional | 50.83292 | -0.97408 | 7.08787 | 1.68646 | 187932.9 |
| Relative Positional  + Frequency-weighted | 50.82480 | -1.02140 | 7.11876 | 1.72649 | 188059.9 |
| Relative Positional  + Frequency-weighted,  + Smoothed | 51.03021 | -1.15668 | 8.26838 | 1.45804 | 188059.9 |
| Absolute Positional  + Smoothed | 52.95626 | -2.64322 | 6.81189 | -2.55959 | 188252.1 |
| Absolute Positional | 52.95591 | -2.64340 | 6.81094 | -2.55890 | 189100.6 |
| Absolute Positional  + Frequency-weighted | 52.99178 | -2.25829 | 6.75389 | -2.48905 | 189668.1 |
| Absolute Positional,  + Frequency-weighted  + Smoothed | 52.99200 | -2.25728 | 6.75381 | -2.48962 | 189668.3 |

**Table 9:** Coefficients and AIC scores of the regression models fit to the Spanish dataset from Mayer and Sundara (in prep).

**B.2.3** **Turkish (Mayer 2024, under review)**

| **Model** | **Intercept** | **Uni. Coef.** | **Bi. Coef.** | **Int. Coef.** | **AIC** |
| --- | --- | --- | --- | --- | --- |
| Relative Positional  + Smoothed | 39.42271 | 6.56583 | 2.46451 | 6.26266 | 159545.6 |
| Relative Positional | 39.16679 | 6.88337 | 1.84632 | 7.46382 | 159581.9 |
| Absolute Positional | 45.03984 | -0.33506 | 11.17251 | -1.29134 | 159628.4 |
| Absolute Positional  + Smoothed | 45.03317 | -0.31176 | 11.13564 | -1.28345 | 159628.8 |

**Table 10:** Coefficients and AIC scores of the regression models fit to Turkish data from Mayer (2024, under review).
